# Challenges and proposed solutions to conducting Alzheimer’s disease psychosis trials

**DOI:** 10.3389/fpsyt.2024.1384176

**Published:** 2024-05-15

**Authors:** Clive Ballard, Pierre Tariot, Maria Soto-Martin, Sanjeev Pathak, I-Yuan Liu

**Affiliations:** ^1^ Institute of Health Research, University of Exeter Medical School, Exeter, United Kingdom; ^2^ Banner Alzheimer’s Institute and University of Arizona College of Medicine, Phoenix, AZ, United States; ^3^ Alzheimer Clinical and Research Centre, Gérontopôle, Toulouse University Hospital, University Hospital Institutes (IHU) HealthAge, Toulouse, France; ^4^ Department of Medical Affairs, Acadia Pharmaceuticals Inc., San Diego, CA, United States

**Keywords:** psychosis, Alzheimer’s disease, Alzheimer’s disease psychosis, atypical antipsychotics, biomarkers, challenges, solutions, clinical trials

## Abstract

Alzheimer’s disease psychosis (ADP) produces a significant burden for patients and their care partners, but at present there are no approved treatments for ADP. The lack of approved treatments may be due to the challenges of conducting clinical trials for this disease. This perspective article discusses distinct challenges and proposed solutions of conducting ADP trials involving seven key areas: (1) methods to reduce the variable and sometimes high rates of placebo response that occur for treatments of neuropsychiatric symptoms; (2) the use of combined or updated criteria that provide a precise, consensus definition of ADP; (3) the use of eligibility criteria to help recruit individuals representative of the larger ADP population and overcome the difficulty of recruiting patients with moderate-to-severe ADP; (4) consideration of multiple perspectives and implementation of technology to reduce the variability in the administration and scoring of neuropsychiatric symptom assessments; (5) the use of clinically appropriate, *a priori–*defined severity thresholds and responder cutoffs; (6) the use of statistical approaches that address absolute effect sizes and a three-tier approach to address the fluctuation of neuropsychiatric symptoms; and (7) the implementation of feasible diagnostic and target-engagement biomarkers as they become available. The goal of these proposed solutions is to improve the evaluation of potential ADP therapies, within the context of randomized, placebo-controlled trials with clinically meaningful endpoints and sustained treatment responses.

## 1 Introduction

Alzheimer’s disease psychosis (ADP) is associated with a significant patient and care partner burden (1). However, in the US there are no approved treatments for this neuropsychiatric symptom (1). Furthermore, few clinical trials have even been attempted due to challenges inherent in conducting trials for regulatory approval in ADP, and the few that have, failed to demonstrate efficacy; this article examines some of those trials failed ADP trials and the objective is to discuss seven specific challenges of conducting ADP trials and propose alternatives to overcome those challenges (**Table 1**).

**Table 1:** Seven challenges associated with ADP trials and proposed solutions for each.

Item	Challenges in ADP trials	Proposed solutions
1	Trials of treatments for neuropsychiatric symptoms are associated with variable, sometimes high, rates of placebo response	Minimize placebo response by having screening and baseline eligibility criteria, employing brief psychosocial therapies prior to randomization, conducting a two-tier trial duration approach, and implementing advantageous trial designs
2	There is a need for precise, consensus diagnostic criteria for ADP	Use combined or updated criteria that provide a precise, consensus definition of ADP
3	It is difficult to recruit patients with moderate-to-severe ADP	Use eligibility criteria that ensure recruitment of a representative ADP population, including those in the nursing home setting
4	Variability in administration of neuropsychiatric symptom assessments can impact trial outcomes	Consider multiple perspectives and implement technology to increase the accuracy of neuropsychiatric symptom assessment
5	It is a challenge to use appropriate severity thresholds and responder cutoffs	Use clinically appropriate, *a priori–*defined severity thresholds and responder cutoffs
6	Trials lack methods to address absolute effect sizes and the fluctuation of neuropsychiatric symptoms	Use statistical approaches that incorporate clinically meaningful metrics, such as absolute effect sizes, and a three-tier approach to address the fluctuation of neuropsychiatric symptoms
7	There is a lack of biomarkers to confirm the presence of psychosis or to serve as an effective target for treatment response	Use diagnostic and target-engagement biomarkers of psychosis in AD

AD, Alzheimer’s disease; ADP, Alzheimer’s dementia psychosis.

## 2 Seven challenges associated with trials in ADP

### 2.1 Trials of treatments for neuropsychiatric symptoms are associated with variable, sometimes high, rates of placebo response

High placebo response rates remain a challenge for trials of treatments for neuropsychiatric symptoms (2). Placebo-group improvements could be driven by multiple factors, such as a response in participants or care partners, a regression to the mean in neuropsychiatric symptoms, or psychosocial factors such as the Hawthorn effect (i.e., awareness of being monitored) (2, 3). Placebo responses can be amplified by using multiple active treatment arms if patients are aware of the trial design because they might appreciate that there are more active treatment opportunities (2). For example, the landmark CATIE-AD (NCT00015548) utilized a four-arm trial design (olanzapine, quetiapine, risperidone, and placebo) and reported moderately elevated placebo-response rates (4). Alternatively, two-arm studies may have lower rates of placebo-response as participants aware of the study design would be less likely to expect an active treatment.

Medical organizations and expert groups recommend nonpharmacological strategies as the preferred first-line treatment for neuropsychiatric symptoms (5). The International Psychogeriatric Association (IPA) suggests that psychosocial interventions occur before, during, and after pharmacotherapy (6). A 2012 meta-analysis found that nonpharmacological interventions reduce behavioral and psychological symptoms of dementia (7). Using nonpharmacological interventions prior to randomization and treatment may exclude patients with less severe disease, who may be more prone to placebo responses (2). Furthermore, these interventions may accelerate resolution of the placebo response, allowing treatment response to be evident earlier (i.e., Week 2 rather than Weeks 4-6). Two examples of trials that successfully utilized this approach were CALM-AD and a study of pimavanserin for ADP (Study 019), which both utilized the brief psychosocial therapy (BPST) prior to pharmacological treatment (8, 9).

### 2.2 Need for a precise, consensus diagnostic criteria for ADP

The development of treatments for ADP has been hindered by the ongoing need for precise, consensus diagnostic criteria for psychosis (10). The *DSM-5* definition distinguishes between major and mild diseases but not between delusions and hallucinations (10, 11). This lack of a distinction between delusions and hallucinations is problematic because diagnostic criteria that capture these symptoms are necessary to accurately define the population of patients with ADP (10). Clinical trial enrollment involves identifying a clinical syndrome (e.g., ADP) via specific clinical criteria and then using rating scales to quantify the severity of the syndrome in an accurate and reproducible manner. As an added challenge, the biological and diagnostic criteria for ADP have changed over time.

### 2.3 It is difficult to recruit patients with moderate-to-severe ADP

It is difficult to recruit an adequate study population, and including patients with less severe ADP (e.g., mild-to-moderate) may make it more challenging for treatments to achieve clinically meaningful improvements in neuropsychiatric symptoms due to floor effects (12). Highlighting the heterogeneity of participant disease severities, a 2014 systematic review and meta-analysis of AD and neuropsychiatric symptoms reported a wide range of participant ages (i.e., mean 73.3–85.6 years) and mental states (i.e., Mini-Mental State Examination [MMSE] scores, 4.5–21.2) (13). In addition, symptoms of psychosis can span both hallucinations (i.e. auditory, visual, tactile) and delusions (i.e., persecutory, referential, religious) (10, 11).

Study 019, which included sites across various UK regions, was successful in enrolling older (mean age, 86 years) nursing-home patients who were frail (baseline mean NPI-NH PS, ≥9.5) with high rates of concomitant medication use (≥82% received ≥5 non-antidementia concomitant medications). In addition, an aripiprazole trial enrolled noninstitutionalized patients with ADP who were elderly (mean age, 81.5 years) and had a minimum disease severity (MMSE score 6–24 and score ≥6 on hallucinations and delusions items of NPI) (14).

### 2.4 Variability in the administration of neuropsychiatric symptom assessments can impact trial outcomes

For ratings of neuropsychiatric symptoms and endpoints, it can be difficult to implement training methodology that results in optimal inter-rater reliability (IRR) thresholds (i.e., intra-class correlation coefficients [ICC] ≥0.9). The care partner’s level of burden and stress and the quality and nature of their relationship with the AD patient can influence their symptom assessments (15). Additionally, care partners often represent a heterogenous mixture of family members and professional healthcare workers (15). Late-stage trials require large participant populations, meaning numerous locations, including sites in cultures with differing views of psychosis (16).

The CATIE-AD trial increased the Brief Psychiatric Rating Scale (BPRS) score reliability by having items scored by a trained clinician and collecting additional information from care partners (17). Furthermore, Study 019 successfully reduced the variability in symptom assessment and achieved high IRR (i.e., ICC values >0.9) by having the primary endpoint scored by a central investigator team of 20 trained sub-investigators and using the novel approach of providing training to participant’s care partners to enable them to act more effectively as informants (9, 18).

### 2.5 It is a challenge to use appropriate severity thresholds and responder cutoffs

It is difficult to formulate *a priori*, clinically informed psychosis measures for severity threshold and treatment responses (19). In the schizophrenia literature, the definition of a treatment response varies from a 20% to 50% reduction in symptoms (19–21). Relative to psychosis and response, it is noteworthy that different psychosis scales likely have different operational characteristics, like response metrics (i.e., 30% reduction), and may not be directly comparable (9, 18, 22).

To analyze data by disease severities, Study 019 included *post hoc* analyses of all patients, as well as those with severe psychosis (NPI-NH PS ≥12) (18). In addition, to assess varying levels of responses, the ADAD trial (NCT00417482) of risperidone defined response as a reduction of ≥30% from baseline on the NPI core score and a score of 1 (very much improved) or 2 (much improved) on the CGI of Change scale for overall psychosis or agitation (23).

### 2.6 Trials lack methods to address absolute effect sizes and the fluctuation of neuropsychiatric symptoms

Hypothesis testing and probability values are critical elements of the scientific method and should be supplemented with measures of absolute effect size, such as the number needed to treat (NNT) and the number needed to harm (NNH) analyses (24). These measures may be more relevant in early- (i.e., phase 1b or 2a) versus late-stage (phase 2b, 3, or 4) trials (25). Another important factor of trial design is that neuropsychiatric symptoms can fluctuate over time; Study 019 reported placebo responses for the outcome measure that fluctuated over the 12-week trial (9). To address these fluctuations, trial designs have assessed sustained response along with longer-duration relapse prevention using randomized-withdrawal (2, 9, 23), the idea being that a sustained response likely represents a more clinically meaningful finding than a single timepoint response.

Various designs have also been employed to capture the treatment effect. An aripiprazole trial assessed acute treatment response at 6 weeks (26), whereas, the ADAD trial of risperidone assessed treatment response at 16 weeks, with a randomized withdrawal to assess relapse at 32 weeks (23). Study 019 addressed the fluctuation of responses by conducting a novel, *post hoc* survival analysis requiring two consecutive improvements (i.e., ≥30% or ≥50) between Baseline and Week 12, which was done to reduce the effect of symptom fluctuation on treatment response (18).

### 2.7 There is a lack of biomarkers to confirm psychosis or serve as an effective target for treatment response

In trials, an ADP diagnosis is made primarily on symptoms, due to a lack of established biomarkers (27). The National Institute on Aging and Alzheimer’s Association (NIA-AA) have developed a research framework for the biological diagnosis of AD, with consideration for the presence of AD pathology (27). Biomarkers may be used to screen for AD pathology with blood-based biomarkers, vascular biomarkers, and confirmatory measures (i.e. cerebrospinal fluid [CSF] and neuroimaging), but many patients have concurrent pathology and advanced age, which present a challenge to some invasive measurements (27). The lack of biomarkers is further complicated by the fact that psychosis symptoms tend to appear in the middle and later stages of AD progression, typically after agitation (28). Currently, the extent to which ADP is associated with specific, measurable biomarkers remains to be determined, and no definitive set of biomarkers has been identified (27).

## 3 Seven proposed solutions to overcome challenges of trials in ADP

### 3.1 Use strategies to minimize placebo response

Investigators can reduce placebo responses through a variety of measures including eligibility criteria, trial duration, and study design. Eligibility requirements can be applied at both the screening and baseline assessments to exclude patients who exhibit improvement between screening and baseline; BPST is designed and optimized to detect such patients. The use of brief psychosocial interventions, prior to treatment randomization, aligns with professional guidelines and may accelerate the resolution of placebo response and enable the detection of treatment effects by Week 2 (5, 8). Performing randomization after the psychosocial interventions (1–2 weeks) allows investigators to exclude patients who respond to a non-pharmacological intervention and consequently would not require pharmacological therapy (2).

Investigators can also implement a two-tier approach that uses both short- and long-term trials to assess efficacy, long-term benefit, and relapse. Short-term trials should be long enough (i.e., 5–6 weeks) to allow for the expected placebo responses to diminish but short enough to limit dropout, optimize tolerability for patients and care partners, and ensure that spontaneous recovery does not confound the outcome (2). Long-term trials should be long enough (i.e., 6–32 weeks) to assess the response duration and relapse rate following medication reductions or discontinuation (2).

Investigators can also implement trial designs with just one or two active treatments to reduce a participant’s expectation of receiving an active treatment (2). They may choose to utilize a randomized discontinuation design where the time to relapse is assessed after a treatment is discontinued (23). Another option is to use the sequential parallel comparative design (SPCD), which was developed to minimize placebo responses (29). Stage 1 is populated with individuals less likely to have a placebo response and involves a drug-placebo comparison (29). Stage 2 conducts a re-randomization of placebo non-responders to drug or placebo to demonstrate the treatment difference in participants with a reduced number of placebo responders (29).

### 3.2 Use combined or updated criteria that provide a precise, consensus definition of ADP

There are multiple existing criteria, which could be used to establish ADP. An earlier aripiprazole trial and a more recent lithium trial both used NPI criteria to define ADP (14, 30, 31). However, Study 019 (9, 18) defined possible/probable AD using the established NINCDS–ADRDA criteria (32) and psychosis using the Jeste and Finkel 2000 criteria (33). Investigators could improve the diagnosis of ADP in future trials by using updated diagnostic criteria such as the latest IPA criteria for psychosis (10, 34). The IPA criteria for psychosis in neurocognitive disorders, which was built on the Jeste and Finkel criteria (10, 33), is widely used to define trial populations and has the advantage of having common, culturally appropriate examples of AD delusions and hallucinations (34).

### 3.3 Use eligibility criteria that ensure recruitment of a representative ADP population

Investigators can recruit a more representative population of older, frailer patients by setting eligibility criteria that specify older age (≥50 years), moderate-to-severe impairment based on MMSE score (≥1 and ≤26) and/or NPI hallucination or delusion item score (≥4 or a combined score of ≥6), and moderate to high levels of concomitant, non-antidementia medications (i.e., 5–10 medications). These criteria should allow for the recruitment of patients in the nursing home setting with greater generalizability of results to more severe patient populations. Given the challenges of recruiting elderly patients with moderate-to-severe ADP in clinical trials, public education is necessary to inform individuals that psychosis is a manifestation of AD and to communicate the importance of participating in ADP clinical trials. These solutions should promote the recruitment of patients with advanced-stage disease, which will increase the generalizability of trial results.

### 3.4 Consider multiple perspectives and implement technology to increase the accuracy of neuropsychiatric symptom assessments

The accuracy of neuropsychiatric symptom assessments can be improved by using central investigator teams, multiple perspectives (i.e. clinicians and care partner informants), IRR measurements, and by leveraging recent technological advances (9, 35–38). To garner multiple perspectives, investigators can incorporate insights from clinicians (i.e., NPI–Clinician rating scale), care partners, and patients and domains for multiple symptoms, such as disinhibition (i.e., NPI-H) (35–37).

Emerging technologies, such as digital assistants to collect near-real-time behavioral data (36) and passive measurement strategies (i.e., geolocation, movement, and physiological parameters) (39) should allow trial investigators to assess participants more often and more objectively. These newer technologies can aid in deploying performance-based cognitive tests, which could eliminate biases associated with subjective, questionnaire-based assessments (40).

### 3.5 Use clinically appropriate, *a priori*–defined severity thresholds and responder cutoffs

Investigators may require patients to meet a threshold of at least moderately severe symptoms at both screening and randomization. Trial outcomes could be improved by including pre-specified subgroup analyses, such as analyses of all patients and those meeting *a priori*–defined criteria of severe psychosis (i.e., NPI-NH PS ≥12) (18). Investigators should also consider using *a priori*–defined responder cutoffs that are large enough (i.e., 30% and/or ≥50% reduction from baseline score) to capture a clinically meaningful response (18). Investigators may also determine the percentage of participants with complete remission of psychotic features.

### 3.6 Use statistical approaches that incorporate clinically meaningful metrics and that address the fluctuation of neuropsychiatric symptoms

Investigators should report absolute effect size using metrics such as Cohen’s *d*, area under the curve, success rate difference, attributable risk, and NNT/NNH (24). NNT and NNH are clinically meaningful because they convey the expected number of patients that would need to be treated to see a response or harm, respectively (24).

Select endpoints should capture the fluctuating course of neuropsychiatric symptoms to increase the power to detect a true treatment effect (22, 41). A three-tier approach to capture the fluctuation of neuropsychiatric symptoms could include the following assessments: (1) acute treatment response (i.e. ≤6 weeks), (2) relapse prevention over longer durations (i.e. ≥26 weeks), and (3) sustained response (≥30% and ≥50%) at two successive timepoints. An advantage of using both *a priori*–defined criteria and sustained response is that they increase the likelihood of capturing clinically meaningful changes (19, 20) and fluctuations in neuropsychiatric symptoms (22, 41, 42).

### 3.7 Use diagnostic and target-engagement biomarkers of psychosis in AD

AD could be established using amyloid and tau blood-based biomarkers (i.e., plasma) and vascular biomarkers during screening and confirmed using CSF and/or positron emission tomography (PET) (43). However, there are currently no widely established ADP biomarkers. Risk genes have been identified and biological differences between psychotic and non-psychotic patients with AD are under evaluation and may emerge as valid biomarkers (28, 44). Biomarkers can also support the mechanism of action of experimental therapies and may help guide dosing decisions; for example, PET imaging of 5HT_2A_ receptor occupancy contributed to the decisions regarding the pimavanserin clinical trial dosage (45). The implementation of reliable biomarkers could also help with elucidating the neurobiological burden associated with a patient’s disease stage, which could enable ADP patient selection by severity thresholds and help to identify patients who may be more likely to respond to treatment.

## 4 Discussion

The relatively few trials in ADP to date have included assessments of aripiprazole, escitalopram, haloperidol, lithium, MK-8189, olanzapine, pimavanserin, quetiapine, and risperidone (9, 17, 30, 31, 46–49). However, none of these agents are approved yet for treatment of ADP. KarXT, a combination of the muscarinic agonist xanomeline and the peripheral anticholinergic trospium, is currently under assessment for treatment of ADP (49). The high rate of failure in drug development for ADP emphasizes the unmet need for improved trial methodology to assess nascent neurotherapies more effectively. The trials and design strategies discussed here represent practicable solutions that could improve future ADP trials and thus, patient outcomes.

The continuing need for precise, consensus diagnostic criteria for ADP has hindered prior clinical trials by limiting their ability to recruit ADP patients in an accurate and reproducible way (10). However, future trials could overcome this issue by combining criteria—using the NINCDS–ADRDA (32) and Jeste and Finkel 2000 criteria (33)—or by using an updated diagnostic criteria such as the latest IPA criteria for psychosis (10, 34), which has culturally appropriate examples of delusions and hallucinations (34).

Investigators may also establish possible or probable AD with a biologically-confirmed diagnosis of AD (43). ADP neurobiological criteria could involve screening (i.e., blood-based biomarkers) and possible confirmatory measures (i.e., CSF and radiological techniques) in select populations (27). Diagnostic specificity with biological criteria allows the exclusion of non-AD participants, who likely have differing etiologies with neuropsychological abnormalities dissimilar to those of AD and who would likely respond discordantly to ADP medications. Also, related to biological markers, is the emerging use of imaging to detect amyloid-related imaging abnormalities (ARIA) in AD patients treated with amyloid-beta monoclonal antibodies (50). We raise this issue as in patients receiving an investigational treatment in conjunction with an amyloid-beta monoclonal antibody treatment–and potentially anti-tau therapies in the future–there is the potential for these novel antibody treatments to alter the symptoms of psychosis either favorably, unfavorably, or both, which could confound the interpretation of the investigational product’s efficacy (50).

Another challenge for ADP trials is the availability and use of reliable endpoints that can demonstrate efficacy by capturing the treatment effect (12, 40). Many of the existing ADP endpoints are not reliable, valid, and/or sensitive enough to capture a clinically meaningful change (40). Numerous endpoints are often deployed, but to identify a set of reliable, valid, and sensitive endpoints for ADP, further studies are necessary to identify the most important aspects of treatment for care partners of, and patients with, ADP. When formulating an endpoint, multiple factors should be considered, including the pharmacotherapy mechanism of action, target population, and selection of relevant and valid cognitive/functional and behavioral domains and related tests and questionnaires (40). Investigators should consider using operational definitions, global ratings in conjunction with specific symptoms (36), composite endpoints (51), and adaptive endpoints (52). The ratings should reflect what clinicians believe is clinically relevant and what patients and care partners think is most meaningful (40).

It can be difficult to recruit a diverse patient population for ADP trials across multiple, international study sites, which is often necessary for trials of neuropsychiatric treatments to recruit large populations with moderate-to-severe disease (17). Furthermore, international sites with variable cultural perceptions of symptoms, like psychosis, could impact trial outcomes (16). It is important to enrich study populations with participants from underrepresented, minority groups, as this is critical to advancing our understanding of the generalizability of trial outcomes (53). However, recruiting underrepresented minorities to AD clinical trials has been challenging historically, and most trial participants have been primarily White. The Study 019 population had low diversity: 93% and 98% of the participants in the pimavanserin and placebo arms, respectively, were White (9). This challenge is more pronounced when study sites are located in regions like the UK, where only 6.4% of individuals aged 65 years or older self-identify as members of ethnic, minority groups or multi ethnicities (54). Purposeful trial designs that consider cultural variations and public education among minority communities surrounding the importance of participating in clinical trials could both help improve ADP trials.

Areas at the forefront of psychiatry include the use of emerging technologies and biomarkers for disease confirmation and target engagement. For future trials, blood-based biomarkers could provide a pragmatic screening tool for AD pathology. Use of the seven solutions proposed here should help to identify safe, effective ADP therapies that meet clinically meaningful endpoints, promote sustained responses, and confer meaningful improvements to patient lives.

## Data availability statement

The original contributions presented in the study are included in the article/supplementary material. Further inquiries can be directed to the corresponding author.

## Ethics statement

Ethical approval was not required for the study involving humans in accordance with the local legislation and institutional requirements. Written informed consent to participate in this study was not required from the participants or the participants’ legal guardians/next of kin in accordance with the national legislation and the institutional requirements.

## Author contributions

CB: Writing – original draft, Writing – review & editing. PT: Writing – original draft, Writing – review & editing. MS-M: Writing – original draft, Writing – review & editing. SP: Writing – original draft, Writing – review & editing. I-YL: Writing – original draft, Writing – review & editing.
